# Recessive dystrophic epidermolysis bullosa results in painful small fibre neuropathy

**DOI:** 10.1093/brain/awx069

**Published:** 2017-03-28

**Authors:** Sofia von Bischhoffshausen, Dinka Ivulic, Paola Alvarez, Victor C. Schuffeneger, Juan Idiaquez, Constanza Fuentes, Pilar Morande, Ignacia Fuentes, Francis Palisson, David L. H. Bennett, Margarita Calvo

**Affiliations:** 1 Facultad de Medicina, Universidad de los Andes, Chile; 2 Departamento de Fisiología, Facultad de Ciencias Biológicas, Pontificia Universidad Católica de Chile, Chile; 3 Departamento de Neurología, Facultad de Medicina, Pontificia Universidad Católica de Chile, Chile; 4 Universidad de Valparaiso, Chile; 5 Fundación DEBRA, Chile; 6 Facultad de Medicina, Clínica Alemana Universidad del Desarrollo, Chile; 7 Centro de Genética y Genómica, Facultad de Medicina, Clínica Alemana Universidad del Desarrollo, Chile; 8 Nuffield Department of clinical neurosciences, University of Oxford, UK; 9 Departamento de Anestesiología, Facultad de Medicina, Pontificia Universidad Católica de Chile, Chile

**Keywords:** small fibre neuropathy, epidermolysis bullosa, neuropathic pain

## Abstract

Small fibres in the skin are vulnerable to damage in metabolic or toxic conditions such as diabetes mellitus or chemotherapy resulting in small fibre neuropathy and associated neuropathic pain. Whether injury to the most distal portion of sensory small fibres due to a primary dermatological disorder can cause neuropathic pain is still unclear. Recessive dystrophic epidermolysis bullosa (RDEB) is a rare condition in which mutations of proteins of the dermo-epidermal junction lead to cycles of blistering followed by regeneration of the skin. Damage is exclusive to the skin and mucous membranes, with no known direct compromise of the nervous system. It is increasingly recognized that most RDEB patients experience daily pain, the aetiology of which is unclear but may include inflammation (in the wounds), musculoskeletal (due to atrophy and retraction scars limiting movement) or neuropathic pain. In this study we investigated the incidence of neuropathic pain and examined the presence of nerve dysfunction in RDEB patients. Around three quarters of patients presented with pain of neuropathic characteristics, which had a length-dependent distribution. Quantitative sensory testing of the foot revealed striking impairments in thermal detection thresholds combined with an increased mechanical pain sensitivity and wind up ratio (temporal summation of noxious mechanical stimuli). Nerve conduction studies showed normal large fibre sensory and motor nerve conduction; however, skin biopsy showed a significant decrease in intraepidermal nerve fibre density. Autonomic nervous system testing revealed no abnormalities in heart rate and blood pressure variability however the sympathetic skin response of the foot was impaired and sweat gland innervation was reduced. We conclude that chronic cutaneous injury can lead to injury and dysfunction of the most distal part of small sensory fibres in a length-dependent distribution resulting in disabling neuropathic pain. These findings also support the use of neuropathic pain screening tools in these patients and treatment algorithms designed to target neuropathic pain.

## Teaser

Recessive dystrophic epidermolysis bullosa is a painful condition characterised by repeated blistering of the skin. Von Bischhoffshausen *et al*. report that in this condition, small sensory fibres in the skin are injured leading to neuropathic pain. These findings support the use of neuropathic pain treatment in recessive dystrophic epidermolysis bullosa.

## Introduction

Small nerve fibres that innervate the skin are especially susceptible to damage and degeneration in several systemic diseases such as diabetes mellitus and toxin exposure. However, whether injury to the most distal portion of sensory small fibres due to a primary dermatological disorder can cause neuropathic pain is still unclear. Epidermolysis bullosa is a group of rare inherited bullous disorders characterized by blister formation in response to minor mechanical trauma (Fine *et al.*, 2014). Based on the site of skin cleavage, epidermolysis bullosa is classified into four major types: epidermolysis bullosa simplex (EBS, cleavage plane within the epidermis), junctional epidermolysis bullosa (JEB, cleavage plane in the lamina lucida), dystrophic epidermolysis bullosa (DEB, cleavage plane below the lamina densa), and Kindler syndrome (multiple cleavage planes) (Fine *et al.*, 2008). DEB is inherited in both an autosomal dominant or DDEB (milder form) and an autosomal recessive manner or RDEB (severe form), both of which result from mutations in the type VII collagen gene (*COL7A1*) (Dang and Murrell, 2008). Type VII collagen is a major component of the anchoring fibril located below the basement membrane in the upper dermis, providing stable dermal–epidermal adhesion (Shinkuma *et al.*, 2011).

Pain is an almost universal feature of epidermolysis bullosa, and its management is central to the wellbeing of patients, especially for those with the more severe types of epidermolysis bullosa (Mellerio *et al.*, 2007; van Scheppingen *et al.*, 2008; Goldschneider *et al.*, 2014). Pain severity varies among the different types of epidermolysis bullosa; severe pain is reported in 14% of patients with all types of epidermolysis bullosa. RDEB is the epidermolysis bullosa type that most commonly presents with pain, with at least 50% of patients suffering intense pain daily, and only 5% of patients reporting to be pain-free (Fine *et al.*, 2004). Pain symptoms in RDEB are often severe and resistant to first-line analgesics. More potent opioid analgesics can be effective against moderate to severe pain, but can produce itch. RDEB patients suffer from chronic itch, which diminishes their quality of life (Snauwaert *et al.,* 2014) such that opioids become a potentially unacceptable treatment for pain control (Goldschneider and Lucky, 2010).

A careful assessment of the different types of pain experienced by the patient is essential in planning therapy and monitoring response. Pain may be inflammatory (blisters and wounds), or musculoskeletal (due to atrophy and retractile scars) (Goldschneider and Lucky, 2010). Anecdotal reports suggest the presence of pain with neuropathic characteristics, which could be alleviated with amitriptyline (Chiu *et al.*, 1999) or gabapentin (Allegaert and Naulaers, 2010). Neuropathic pain is defined as pain arising as a direct consequence of a lesion or disease affecting the somatosensory system (Treede *et al.*, 2008). In RDEB the somatosensory system is thought to be intact; however, it could be postulated that chronic blistering and inflammation in the skin could lead to damage to the most distal portion of sensory small fibres.

We hypothesized that pain in RDEB could have a significant neuropathic component if chronic skin inflammation leads to cutaneous small fibre dysfunction. We therefore performed detailed somatosensory phenotyping in the largest cohort yet collected for this purpose including the use of: neuropathic pain screening questionnaires, quantitative sensory testing, autonomic function testing, nerve conduction studies, and morphological assessment of unmyelinated sensory afferents in the skin. Furthermore, potential associations of these outcomes with severity of the disease were determined. Here we provide evidence for the first time of functional and morphological impairment of small sensory fibres in patients with RDEB.

## Materials and methods

### Participants

Patients were recruited from DEBRA (Dystrophic Epidermolysis Bullosa Research Association) Chile. Although epidermolysis bullosa is a rare disease (in Chile the overall incidence of epidermolysis bullosa is 19.6 new cases per million live births; DEBRA Chile, unpublished data), DEBRA Chile cares for 64 patients with RDEB, which made this project feasible. Patients with clinical and molecular diagnosis of RDEB (with confirmed mutations in the *COL7A1* gene) were asked by their physician for permission to be contacted for participation in the study. Those who accepted were asked to fill in questionnaires and to attend a single appointment during which one trained examiner performed electrodiagnostic tests, quantitative sensory testing (QST), autonomic tests, and a skin biopsy. We only included patients that were over 13 years old, because in performing QST a minimum understanding of instructions is required and in this specific cohort of patients cognition is limited by low educational level due to poor school attendance. The study was approved by the Institutional Ethics Committee of Pontificia Universidad Católica de Chile (reference number 13-317), and all participants gave informed written consent before participating. The study was conducted in accordance with the Declaration of Helsinki.

### Symptom and function questionnaires

RDEB can differ in its presentation, so to determine the severity of clinical manifestations and the disease impact of the symptoms we used the Birmingham Epidermolysis Bullosa Severity (BEBS) score (Moss *et al.*, 2009). This scoring system is simple and reliable and includes the following items: area of damaged skin, involvement of nails, mouth, eyes, larynx and oesophagus, scarring of hands, skin cancer, chronic wounds, alopecia and nutritional compromise.

We used the DN4 questionnaire (Douleur Neuropathique en 4 Questions; a screening tool for neuropathic pain consisting of questions on symptoms and a brief physical test). At the cut-off of 4, DN4 has sensitivity of 80%, and specificity of 92% (Bouhassira *et al.*, 2005; Spallone *et al.*, 2012) in identifying neuropathic pain and has been validated in Spanish (Perez *et al.*, 2007). The Neuropathic Pain Symptom Inventory (NPSI) is a self-administered questionnaire that evaluates the presence and severity of 10 different neuropathic pain descriptors, each on an 11-point scale where 0 indicates no symptoms and 10 indicates maximal symptoms experienced (Bouhassira *et al.*, 2004). This questionnaire has been validated in Spanish (Villoria *et al.*, 2011).

### Structured neurological examination

A comprehensive structured upper and lower limb neurological examination was used to detect clinical signs of a peripheral neuropathy (Phillips *et al.*, 2014). The examination was performed on each patient and included assessment of deep-tendon reflexes, muscle wasting and power as well as sensory response to light touch, cold, and pinprick sensation, vibration and proprioceptive function.

#### Quantitative sensory testing

Sensory profiles were determined according to the protocol of the German Research Network on Neuropathic Pain (DFNS) (Rolke *et al.*, 2006). The DFNS has developed and validated a comprehensive QST battery, which uses standardized equipment, paradigms, and verbal instructions as described. The investigator (M.C.) underwent a formal course of instruction in conducting the DFNS QST protocol at BG University Hospital, Bochum. Cold and warm detection thresholds as well as cold and heat pain thresholds and thermal sensory limen were established using the TSA-II – NeuroSensory Analyzer (Medoc). We tested mechanical detection using optic glass fibres Von Frey filaments (OptiHair MARSTOCKnervtest). Mechanical pain thresholds, mechanical pain sensitivity, and wind up ratio were measured using PinPrick stimulators (MRC). The pressure pain thresholds were measured using an algometer (FPK10, Wagner Instruments), and the vibration detection threshold using a Rydel–Seiffer graded tuning fork (64 Hz, 8/8 scale, YNR). Participants were familiarized with the testing procedure on the dorsum of the arm before all parameters were measured over the dorsum of the foot (S1 dermatome). Pressure pain thresholds were recorded in the foot instep, vibration detection thresholds were recorded over a bony prominence (malleolus internus). QST data entry was into an Excel-based (Excel 2007; Microsoft) data analysis system (Equista) provided by the DFNS. Based on the log transformed raw values for each QST item a z-score sensory profile was calculated: z-score = (value of the subject − mean value of control subjects) / standard deviation of control subjects. Positive z-scores represent gain of function whereas negative z-scores denote loss of function. For individual analysis values were compared with published reference data (Rolke *et al.*, 2006). For group analysis patients’ data were compared with values of age- and gender-matched healthy control subjects from our laboratory.

### Nerve conduction studies

Nerve conduction studies were performed using an ADVANCE system (Neurometrix) with conventional reusable electrodes (Natus Neurology). The foot was warmed to ensure a temperature of 32°C. We recorded nerve action potential amplitudes and nerve conduction velocities from the sural nerve (sensory) and the peroneal nerve (motor). For the sural nerve the active recording electrode was placed behind the lateral malleolus with the reference electrode distal, and the stimulation point was placed at 14 cm proximal to the active electrode in the midline of the posterior lower leg. For the peroneal nerve the active electrode was placed over the midpoint of the extensor digitorum brevis muscle on the dorsum of the foot, and the reference electrode was placed slightly distal to the fifth metatarsophalangeal joint. Stimulation points were: (i) 8 cm proximal to the active electrode, slightly lateral to the tibialis anterior tendon; and (ii) slightly posterior and inferior to the fibular head.

Nerve conduction studies could not be recorded from patients that presented with active wounds at a recording or stimulating site.

### Testing of autonomic function

#### Blood pressure and heart rate response to standing

Lying and standing blood pressure and heart rate was measured using a DINAMAP monitor (Critikon). Lying blood pressure and heart rate were measured first, after which the subject was asked to stand for 10 min to measure standing blood pressure and heart rate at 1 and 10 min. Orthostatic hypotension was determined to be present in subjects in whom either at least a 20 mm Hg reduction in systolic or a 10 mm Hg reduction in diastolic blood pressure was observed.

### Heart rate variability

#### Heart rate response to deep breathing

The patient was connected to an electrocardiograph monitor. The test was performed with the patient lying quietly and breathing deeply at six breaths per minute (inspiratory and expiratory cycles of 10 s), during five successive breathing cycles. The maximum–minimum heart rate during each breathing cycle was measured and the mean of the differences was calculated.

#### Valsalva ratio

Subjects were asked to perform a forced expiration (40 mmHg) during 15 s. The ratio of the maximal heart rate generated during the Valsalva manoeuvre divided by the lowest heart rate following the manoeuver was calculated.

#### Sympathetic skin responses

Sympathetic skin responses were recorded using EMG equipment (Cadwell Sierra Wave). The differential surface electrodes were fixed at the sole of the right foot with the reference electrode fixed on the dorsum of the foot. Responses were recorded in a quiet dimly lit room at 22–24°C, with the subject supine and relaxed, with the skin temperature at 32–36°C. Responses were elicited by asking the subject to do deep breathing. The recording time was 10 s, the lower frequency limit was 0.1 Hz, and the upper limit 200 Hz. Amplitude of the sympathetic skin response was analysed determining the peak-to-peak distance.

#### Quantification of intraepidermal nerve fibre density

Skin biopsy was performed using a 3-mm disposable punch under sterile technique, after topical anaesthesia with lidocaine on the distal part of the leg (10 cm above the lateral malleolus), following published guidelines (Lauria *et al.*, 2010). The skin biopsy was only taken from skin where there was no active blistering. The biopsy was fixed in fresh paraformaldehyde (4%) for 2–4 h. Tissue was then washed in 0.1 M phosphate buffer and stored for 2–3 days in 20% sucrose in 0. 1 M phosphate buffer. After embedding in O.C.T., the tissue was snap frozen and stored at −80°C. Sections were cut in a cryostat at 50 μm, were blocked with 5% fish gelatine for 1 h, and were incubated overnight at 4°C with an antibody against protein gene product 9.5 (PGP 9.5, Ultraclone, 1:1000). The next day, sections were washed in PBS containing 0.1% Triton^TM^ X-100 and secondary antibody was incubated overnight at 4°C (anti-rabbit Cy3 Stratech, 1:1000). On the third day, sections were washed and mounted for analysis. We used immunofluorescence instead of bright field microscopy because we have more experience with the former technique, and both have shown to have comparable diagnostic efficiency (Nolano *et al.*, 2015). Intraepidermal nerve fibre density (IENFD) was determined by the same blinded observer on three sections per participant using a Zeiss LSM Pascal 5 (Carl Zeiss) connected to an inverted microscope (Axiovert 2000) using a 40× objective. Epidermal fibres that crossed the dermal–epidermal junction were counted, whereas secondary branches and fragments were excluded from quantification. The length of the epidermal surface was measured using ImageJ (http://imagej.nih.gov) and IENFD was expressed as fibres per mm epidermis. The investigator performing this technique (M.C.) trained at a well-established skin biopsy laboratory (Prof David Bennett at Oxford University). To calculate z-scores we used normative data on IENFD done with immunofluorescence (Provitera *et al.*, 2016). Z-scores were calculated using the mean (μ), and the standard deviation (σ) for each age range and sex [z = (x − μ) / σ] (Provitera *et al.*, 2016).

#### Quantification of dermal innervation

Skin section were immunostained with PGP9.5, CD31 to label endothelial cells (1:100, Dako, clone JC70A), and DAPI following the protocol described above. Images were taken at the same exposure conditions in a confocal microscope (Nikon SPECTRAL ECLIPSE C2) using a 60× objective. Dermal innervation was quantified measuring immunofluoresence intensity in ImageJ. Sweat glands were identified by their tubular structure seen by DAPI, and their innervation was quantified by measuring PGP9.5 immunostaining signal over them. Labelling endothelial cells with CD31 identified blood vessels, and their innervation was quantified by measuring PGP9.5 immunostaining signal over them. For each analysis we examined three randomly chosen sections per subject (each of which contained two to three sweat glands, and two to three blood vessels).

### Statistical analysis

SPSS Statistics Version 23 (IBM) was used for statistical analysis. Data were tested for normality with the Kolmogorov-Smirnov test. Mean values and standard error of the mean (SEM) are reported for normally distributed data. QST z-scores, results from nerve conduction studies, autonomic tests, and skin biopsies were compared with independent samples *t*-tests or Mann-Whitney U-tests as appropriate. Pearson’s correlation analyses were performed to explore associations between QST findings, IENFD and severity of the disease (BEBS).

## Results

### Patients

DEBRA Chile cares for 64 RDEB patients of whom 36 are over 13 years of age. We prospectively enrolled 29 of these patients. From the remaining seven patients: three were excluded because they presented cognitive impairment (one with Down’s syndrome, one with acute psychosis, and one due to illicit drug use), and four declined to participate in the study. An age and gender matched cohort of 27 healthy volunteers without any dermatological, neurological or systemic medical conditions were recruited from the general population in Santiago. The mean age of patients was 22.3 ± 12.1 years [standard deviation (SD)] and of controls was 26.5 ± 7.9 years (SD), with no significant difference between both groups (*P* = 0.13). There were more females in the RDEB group than in the control group but this was not statistically significant (*P = *0.3). Demographic data are shown in Table 1. All patients had clinical and molecular diagnosis of RDEB (classification according to Fine *et al.*, 2014) with confirmed mutations in the *COL7A1* gene. The severity of RDEB can vary between patients, so we used the BEBS score (Moss *et al.*, 2009) to assess disease severity. This scoring system measures different complications of epidermolysis bullosa such as: area of skin involvement, nail dystrophy, mucosal involvement (eyes, mouth, larynx, oesophagus), scarring of hands, skin cancer, chronic wounds present for >6 months, alopecia, and nutritional compromise. The mean score was 36.8 with an SD of 19.6. The majority of patients therefore had mild/moderate disease and none had a score of >90, which would be a severe category. As an example of typical symptoms we have given a typical case report in the Supplementary material.

**Table 1:** Demographic features of RDEB patients and healthy control subjects

	Controls	RDEB	*P*-value
***n***	27	29	
**Sex, *n* (%)**			0.3
**Female**	8 (29.6)	16 (55.2)	
**Male**	19 (70.4)	13 (44.9)	
**Mean age, years (SD)**	26.5 (7.9)	22.3 (12.1)	0.13
**Mean pain NRS (SD)**	0 (0)	4.2 (0.52)	>0.001

Data are presented as mean ± (SD). NRS = numerical rate scale.

### A high prevalence of pain with neuropathic characteristics in RDEB

Most RDEB patients (92.8%) interviewed said they have pain every day, with a mean rating of 4.2 ± 0.52 in the numerical rating scale (0–10). Pain starts in early life, as blistering develops at birth or very shortly after birth. Acute pain occurs localized to areas of active skin blistering and resolves following healing. However, in addition patients also describe persistent pain arising from uninjured areas of skin and especially localized to the feet. We asked for the duration of this pain that was unrelated to active wounds, and found that it varied significantly ranging from 1 to 25 years with a mean duration of 4.7 years (SD: 5.1 years).

Only a minority of RDEB patients took regular analgesic medications, although all of them complained of chronic pain. These included paracetamol (20%), ibuprofen (3.4%), tramadol (10%), pregabalin (3.4%) and amitriptyline (3.4%). Some patients were prescribed medication for other conditions such as depression, anxiety disorders, and epilepsy (duloxetine 3.4%, sertraline 3.4%, zolpidem 3.4%, clonazepam 3.4%, and carbamazepine 3.4%, respectively). Of the control subjects, four subjects took antidepressants (sertraline 11%, fluoxetine 3.7%).

We assessed the presence of specific descriptors of pain by using two complementary questionnaires to understand if the pain reported by RDEB patients had neuropathic characteristics. We used the DN4 as a screening tool for neuropathic pain and found that 75.9% of RBED patients had a score of 4 or higher, which is highly suggestive of neuropathic pain (Bouhassira *et al.*, 2005; Spallone *et al.*, 2012). The mean DN4 score was 5.03 ± 0.43. We used a body map to define pain location. In all cases pain was principally localized to the legs and had a length-dependent distribution with particular involvement of the feet: 25% of patients had pain only in the feet, 50% had pain from the toes up to the knees, and 25% had pain from the toes up to the hip. We also used the Neuropathic Pain Symptoms Questionnaire (NPSI; Bouhassira *et al.*, 2004), a self-administered questionnaire specifically designed to evaluate the different symptoms of neuropathic pain. It revealed that 60% of patients had burning sensations, 56% had tingling sensations, 52% had electric shock sensations, 48% had pin and needles, and 72% had stabbing sensations of moderate or severe intensity. These pain descriptors have been shown to be expressed preferentially in patients with neuropathic pain and have a discriminant value (Bouhassira *et al.*, 2005).

### Clinical examination findings in patients with RDEB are consistent with small fibre neuropathy

Clinical findings were consistent with small fibre neuropathy. Muscle wasting was difficult to assess due to the degree of deformity from tissue fibrosis. There was no weakness. In certain cases, deep tendon reflexes at the ankle could not be assessed due to soft tissue contractures of the extremities. In all cases the patellar reflex was present and normal. Large fibre sensory function including light touch, vibration and proprioception was normal. Pin prick sensibility was impaired only in one patient. Thermal sensibility to cool was impaired in 80% of cases in a distal to proximal gradient (impaired up to the metatarsophalangeal joints in 20%, up to the ankle in 16%, up to the knees in 40%, and up to the hip in 4%). In a minority of cases (16%) thermal sensitivity was also impaired in the hands (Supplementary Table 1).

#### Quantitative sensory testing in patients with RDEB reveals a deficit in thermal detection thresholds

**Figure 1:**
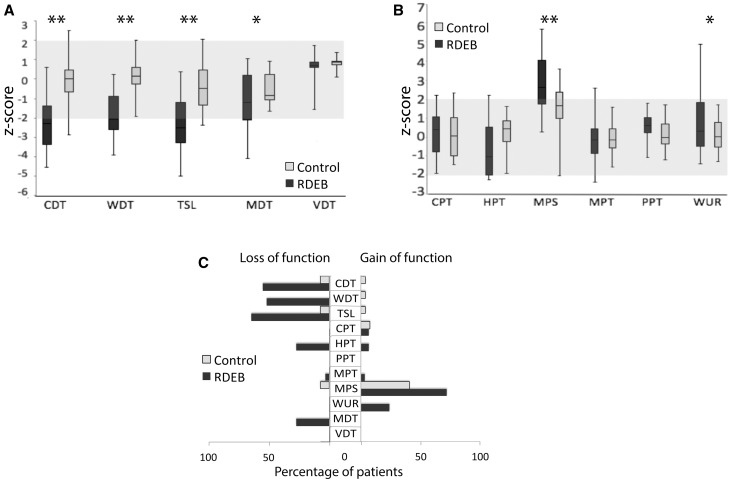
**Somatosensory profiles.** Somatosensory profiles determined with QST in the dorsum of the foot (S1 dermatome) of patients with RDEB (dark grey) and healthy volunteers (light grey). Data are expressed as mean z-scores with standard deviations and the grey area indicates the normal range of ±2 SD of normative data. (**A**) Patients with RDEB have a significant loss of function in cold and warm detection thresholds compared to control subjects. They also have a reduced ability to differentiate temperature changes (e.g. thermal sensory limen). A small but significant decrease in mechanical detection thresholds was observed, but no patients present loss or gain in vibration detection thresholds. (**B**) RDEB patients had increased pain sensibility compared to control participants, which could be seen as an altered heat pain sensitivity, altered mechanical pain sensitivity and altered wind up ratio. No differences in cold pain thresholds were found between both groups. There was also no difference in mechanical and pressure pain thresholds. (**C**) Percentage of patients with value outside the normal range. To the left side are shown the loss-of-function (values < −2). To the right side are shown the gain of function (values > 2). *P < 0.05, **P < 0.001. CDT = cold detection threshold; CPT = cold pain threshold; HPT = heat pain threshold; MDT = mechanical detection threshold; MPS = mechanical pain sensitivity; MPT = mechanical pain threshold; PPT = pressure pain threshold; TSL = thermal sensory limen; VDT = vibration detection threshold; WDT = warm detection threshold; WUR = wind up ratio.

QST over the dorsum of the foot (S1 dermatome), revealed that patients with RDEB had significantly reduced cold and elevated warm detection thresholds (i.e. reduced sensitivity to warm and cool stimuli) as well as increased thermal sensory limen compared to control participants (all *P* < 0.0001, Fig. 1A). This is indicative of loss of function mediated by C and Aδ fibres. One-third of the patients also showed a small but significant decrease in mechanical detection thresholds (*P* = 0.02), which is indicative of loss of low threshold mechanosensation mediated by β fibres. No patient presented with loss or gain in vibration detection thresholds (*P = *0.13) (Fig. 1A).

RDEB patients also had some evidence of gain-of-function compared to control participants including: decreased heat pain thresholds (*P = *0.02), increased mechanical pain sensitivity (*P* < 0.001), and increased wind up ratio (*P = *0.02) (Fig. 1B). No differences in cold pain thresholds (*P = *0.53), mechanical pain thresholds (*P = *0.86), and pressure pain thresholds (*P = *0.14) were found between both groups (Fig. 1B). We looked at the percentage of patients with QST values outside the normal range and plotted Fig. 1C to illustrate frequency of loss-of-function (z-values < −2) and gain-of-function (z-values > 2) in patients and controls.

Pressure pain thresholds could not be measured in eight patients because of concern regarding exacerbation of blistering and wounding of the skin. One-third of RDEB patients presented with abnormal paradoxical heat sensations (a sensation of warmth on skin cooling) and 17.2% of them demonstrated dynamic mechanical allodynia phenomena not observed in controls. Overall, the somatosensory phenotype of RDEB patients reflects hypoaesthesia in small fibre mediated domains but in addition, a number of examples of hypersensitivity including mechanical and thermal hyperalgesia and brush evoked allodynia.

### Sensory profile changes in patients with RDEB correlate with the severity of the disease

**Figure 2:**
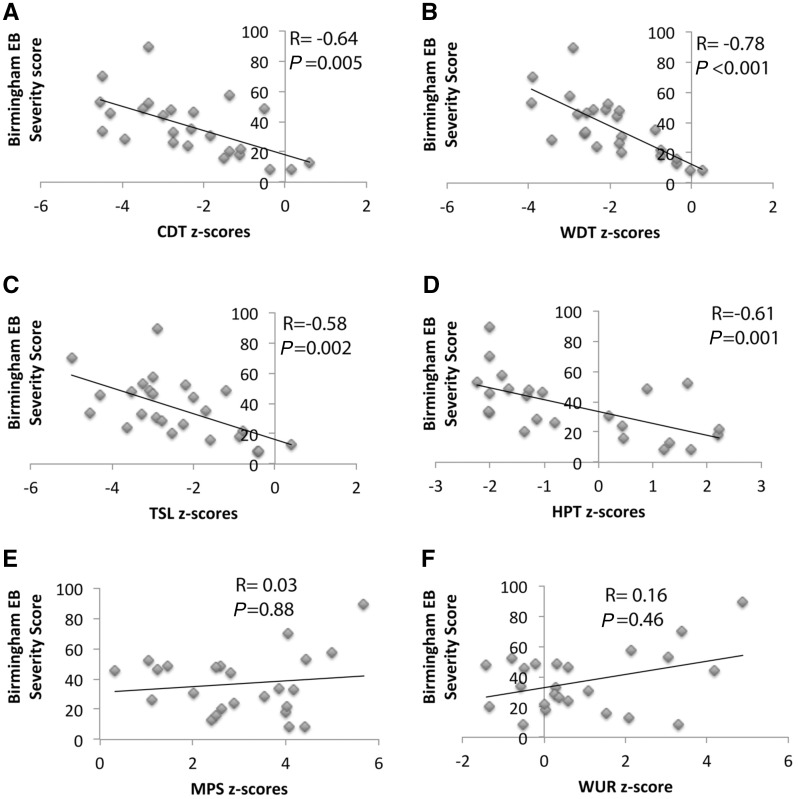
**Correlation of QST findings and severity of RDEB disease.** Pearson’s correlation analyses were performed to explore associations between findings on the quantitative sensory profile and the severity of RDEB measured using the Birmingham severity score (BEBS). (**A**) The correlation coefficient (r = −0.64) showed that there was a negative correlation between cold detection threshold (CDT) and severity of RDEB disease. This correlation was highly significant (*P* = 0.005), *n* = 29 patients. (**B**) The correlation coefficient (r = −0.78) showed that there was a negative correlation between warm detection threshold (WDT) and severity of RDEB disease. This correlation was highly significant (*P* < 0.001), *n* = 29 patients. (**C**) The correlation coefficient (r = −0.58) showed that there was a negative correlation between thermal sensory limen (TSL) and severity of RDEB disease. This correlation was highly significant (*P* = 0.002), *n* = 29 patients. (**D**) The correlation coefficient (r = −0.61) showed that there was a negative correlation between heat pain threshold (HPT) and severity of RDEB disease. This correlation was highly significant (*P* = 0.001), *n* = 29 patients. (**E** and **F**) There was no significant correlation between the mechanical pain sensitivity and the wind up ratio and the severity of disease (r = 0.003, r = 0.16, respectively) *n* = 29 patients.

The level of chronic damage produced in the skin depends on the severity of RDEB, and we hypothesized that this would also be reflected in the degree of small fibre injury. We investigated the correlation between severity of disease (using the BEBS score) and the different QST parameters in which we found significant loss- or gain-of-function in patients with RDEB (Fig. 2). We found that loss in cold and warm detection thresholds and thermal sensory limen inversely correlated with RDEB severity score (Fig. 2A–C). We observed that severity of disease in RDEB patients also correlates with lower heat pain thresholds (Fig. 2D). There was a small trend for an inverse correlation for mechanical detection threshold, which was not significant (r = −0.28, *P = *0.16). For the tests that demonstrated a gain-of-function in RDEB patients (mechanical pain sensitivity and wind up ratio) we found no correlation with the RDEB severity scores (Fig. 2E and F).

The BEBS score consists of eight items: area of damaged skin, involvement of nails, involvement of mucous membrane, scarring of hands, skin cancer, chronic wounds, alopecia and nutritional compromise. We investigated the correlation of the different items of the BEBS with the different measurements of the QST that correlated with BEBS (note that skin cancer was excluded as no patients had developed skin cancer). We found that scarring of the hands correlates very well and consistently with loss-of-function on thermal tests (cold detection threshold, warm detection threshold, thermal sensory limen, and heat pain threshold). Scarring of the hands gets worse with the repetitive cycles of blistering and regeneration and therefore reflects active and long-lasting disease affecting the hands (and probably the lower limbs as well) (Supplementary Table 2).

Therefore, small sensory afferents (C and Aδ fibres) seem to be the most affected by the level of damage produced in the skin by the disease.

#### Neurophysiology of sural and peroneal nerves were normal in RDEB patients

Nerve conduction studies were done in 21 volunteers and 21 RDEB patients. Neurophysiology could not be undertaken in some RDEB patients where the location of active wounds made it impossible to perform the tests or the investigation was declined. Sensory and motor conduction examined in the sural and peroneal nerves revealed no difference in any parameters comparing patients with RDEB and controls (Table 2).

**Table 2:** Neurophysiology data of RDEB patients and healthy control volunteers

	Sural nerve		Peroneal nerve	
	SNAP (µV)	NCV (m/s)	CMAP (mV)	NCV (m/s)
Controls (*n* = 21)	22.3 ± 1.6	51.1 ± 1.6	3.4 ± 0.4	52.3 ± 1.9
RDEB (*n* = 21)	18.5 ± 1.9	51.2 ± 1.5	3.2 ± 0.5	50.2 ± 1.7
*P*-value	0.12	0.9	0.8	0.7

CMAP = compound muscle action potential; NCV = nerve conduction velocity; SNAP = sensory nerve action potential.

#### RDEB patients have histological evidence of intra-epidermal small fibre loss

**Figure 3:**
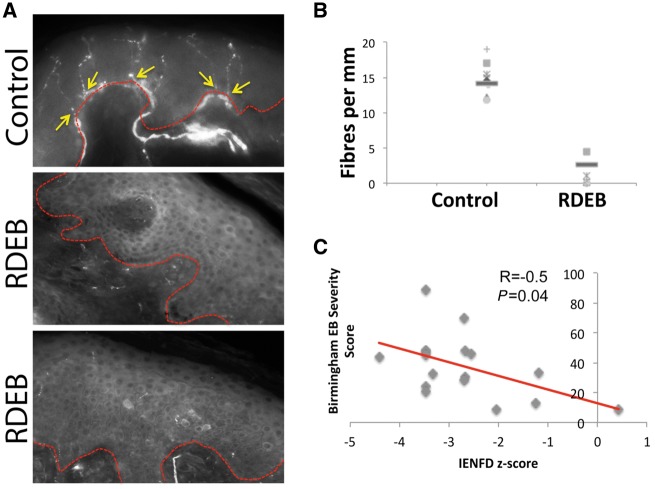
**RDEB patients have a reduced IENFD.** (**A**) Representative sections of skin biopsy of two RDEB patients and an age matched control volunteer immunostained with PGP9.5 (a pan neuronal marker). The dashed red line indicates the limit between dermis and epidermis. In the control subject it can be seen that there are several thin fibres crossing the dermo-epidermal border (yellow arrow). Conversely, the skin of RDEB patients shows very few, if any, fibres crossing into the epidermis. Note that there is no active skin blistering observable at the site of the biopsy. Scale bar = 100 μm. (**B**) The graph shows the mean IENFD expressed as fibres per mm as well as single data points of every subject. RDEB patients have a significantly lower IENFD than the matched control group (*P* < 0.001). Note that in **A** and **B** the controls subjects are from our own Chilean cohort. (**C**) Pearson’s correlation analysis was performed to explore the association between IENFD and the severity of RDEB measured using the BEBS sore. There was a negative correlation coefficient of r   =  −0.5.This correlation was significant (*P* = 0.04), *n*   = 18 patients. In this panel, z-scores were generated using published normative data (Provitera *et al.*, 2016).

Skin biopsies to quantify intraepidermal nerve fibre density in the distal leg were performed in 18 volunteers and 18 RDEB patients and were only taken from sites in which there was no active blistering. There was a striking reduction in IENFD in RDEB patients [mean (SD) fibres per mm: 2.6 (1.2)] compared to our controls recruited in Chile [14.1 (0.6), *P < *0.0001, Fig. 3A and B], implying a severe loss of small nerve fibres. Only one RDEB patient had a value above the gender/age-adjusted IENFD 5° quintile cut-off when comparing to published normative data generated from an Italian cohort (Provitera *et al.*, 2016). In comparison, all of our control subjects were above this cut-off value. To investigate if the severity of the disease correlates with the loss in IENFD, we calculated the z-score of each patient compared to these normative values (Provitera *et al.*, 2016) to correct for age and gender differences. We found that z-scores of IENFD were inversely dependent of the severity of the disease, as measured by the BEBS score (r = −0.5, *P = *0.04, Fig. 3C). We quantified dermal innervation and found no difference in PGP9.5 immunofluoresence signal between RDEB patients and controls (*P* = 0.3, Supplementary Fig. 2)

#### Assessment of autonomic function

We also assessed autonomic nervous system function. Tests that assess blood pressure responses to orthostatic testing are in a large part a reflection of sympathetic activity. Conversely, changes in heart rate during orthostatic testing and Valsalva manoeuvre, as well as during deep breathing, reflect parasympathetic modulation (Jaradeh and Prieto, 2003; Mathias, 2003; Zygmunt and Stanczyk, 2010). We assessed the blood pressure response to standing and found that at rest, 1 and 10 min after standing, there were no significant difference between RDEB patients and healthy volunteers (Table 3). We observed a statistically significant difference in the baseline heart rate between controls and RDEB patients, which can be explained by the anaemia often seen in patients due to malnutrition and blood loss through extensive wounds (Mellerio *et al.*, 2007). In our cohort the mean haemoglobin of male patients was 93 ± 29 g/l, and of female patients was 97 ± 19 g/l. Even though there was this baseline difference, the heart rate of RDEB patients varied at 1 and 10 min after standing in the same way as it did in healthy volunteers. Similarly, no statistical difference was observed in heart rate variability induced by Valsalva or by deep breathing (Table 4). These data indicate that in RDEB patients there is no systemic dysfunction of autonomic C fibres modulating cardiovascular function.

**Table 3:** Assessment of autonomic function

	Controls (*n = *10)	RDEB (*n = *11)	*P*-value
**Basal**	100/62 ± 12/7	101/53 ± 12/11	S = 0.8, D = 0.1
**1 min**	105/69 ± 10/5	109/61 ± 16/8	S = 0.6, D = 0.2
**10 min**	109/72 ± 10/8	104/60 ± 15/6	S = 0.5, D = 0.2

Blood pressure in response to standing in RDEB patients and in healthy volunteers. At rest (basal), 1 and 10 min after standing, there were no significant difference related with blood pressure response between RDEB patients and controls. D = diastolic blood pressure; S = systolic blood pressure.

**Table 4:** Assessment of autonomic function, heart rate

	Controls (*n = *10)	RDEB (*n = *11)	*P*-value
HR ratio induced by standing 1 min	1.09 ± 0.04	1.1 ± 0.3	0.7
HR ratio induced by standing 10 min	1.19 ± 0.04	1.1 ± 0.06	0.2
HR ratio induced by Valsalva	1.33 ± 0.1	1.33 ± 0.2	0.9
HR response to deep breathing (Δmax–min)	17.5 ± 4	16.7 ± 11	0.8

Heart rate (HR) responses to orthostatic testing, Valsalva manoeuvre, and deep breathing. No statistical difference was observed between RDEB patients and controls related with heart rate variations induced by any of the tests.

**Figure 4:**
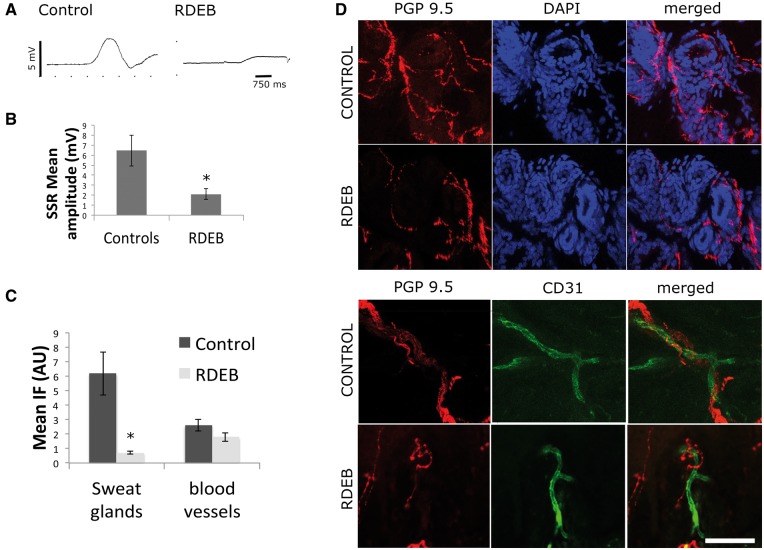
**Sympathetic skin response and autonomic innervation.** The sympathetic skin response measures changes in skin conductance, which depends on the presence of sweat. Sweating is controlled by the sympathetic nervous system, and therefore skin conductance is a surrogate measure of activity of the sympathetic system. In **A**, representative traces of sympathetic skin response are shown (control and RDEB, respectively). Responses were elicited by suddenly asking the subject to take a deep breath. In **B** we show the mean sympathetic skin response amplitude of control subjects and RDEB patients. In **C** we show quantification of PGP 9.5 immunofluorescence signal in sweat glands and in blood vessels in RDEB and control subjects. In **D** representatives sections of dermis of control and RDEB are shown. These sections were immunostained with CD31 to label endothelial cells (green), PGP9.5 to label nerves (red), and DAPI to show nuclei and the tubular structure of sweat glands (blue) (*n* = 10 per group, scale bar = 30 μm).

We then tested the autonomic C fibres in the skin, which are susceptible of injury due to skin damage. We measured the sympathetic skin response that results from reflex activation of the sudomotor sympathetic efferent fibres, which induces changes in skin resistance to electrical conduction. We found that sympathetic skin response amplitudes were significantly lower in RDEB than in controls (*P* = 0.02). Moreover, sympathetic skin response was absent in two patients with RDEB and was present in all control subjects (Fig. 4A–C)

We investigated sweat glands and blood vessels innervation in the dermis using immunofluorescence. We found that sweat gland innervation was reduced while blood vessels innervation remained the same (Fig. 4D)

#### Presence of neuropathic pain in RDEB patients using the IASP NeuPSIG grading system

Because of the lack of a specific diagnostic tool for neuropathic pain, a grading system of definite, probable, and possible neuropathic pain has been proposed and recently updated (Treede *et al.*, 2008; Finnerup *et al.*, 2016). We applied this updated grading system to our patients: history of pain in plausible anatomical distribution (in this case length dependant neuropathy) with a known diagnosis of RDEB means that neuropathic pain is possible. Finding sensory deficits/gain of function using clinical examination and/or QST means that neuropathic pain is probable. And confirmation of reduced IENFD means that neuropathic pain is definite. Sixty-two per cent of patients with RDEB had a definite diagnosis of neuropathic pain, 24% had a probable diagnosis of neuropathic pain, and 13.7% did not report pain with a plausible anatomical distribution. It is worth noting that patients with a probable diagnosis did not reach the definite criteria because we were unable to perform the confirmatory test, in this case a skin biopsy.

Using this grading system as a gold standard (Finnerup *et al.*, 2016), we found that most patients with a definite or probable diagnosis of neuropathic pain scored over the 4 points cut-off in the DN4 screening tool (72% and 85%, respectively). Applying DN4 to this RDEB cohort we found the sensitivity to be 81.5% and specificity to be 73% for the diagnosis of neuropathic pain.

## Discussion

In this study we show for the first time that patients with RDEB have a high rate of neuropathic pain due to a small fibre neuropathy. Chronic skin damage as a consequence of RDEB appears to exclusively affect the unmyelinated/thinly myelinated sensory and autonomic nerve fibres that innervate the skin. The functional impairment and reduction in IENFD correlates with the severity of this dermatological condition. Neurons that do not have axons transiting or very close to the dermo-epidermal boundary (large sensory and motor fibres) and as such are less exposed to the damage and healing processes within the skin, are not affected. These results suggest that neuropathy is caused by repeated episodes of blistering and skin regeneration and emphasize the need for adopting treatment algorithms for neuropathic pain in these patients.

### Detecting neuropathic pain in RDEB

Chronic pain in RDEB patients is a common and debilitating problem and in accordance with previous literature we found that in our cohort, over 90% of patients have daily moderate-to-severe pain (Fine *et al.*, 2004). Chronic pain management is often complex in RDEB patients due to the mixed aetiologies of pain (Goldschneider and Lucky, 2010). It is important to recognize if there is any neuropathic component in each patient’s pain because it will lead to a specific treatment (Goldschneider *et al.*, 2014). Anecdotal reports point out that patients use specific words to describe their pain that are suggestive of neuropathy such as burning pain (Fine *et al.*, 2004). To further investigate this, we used two complementary questionnaires to directly ask for pain descriptors that have been shown to be expressed preferentially in neuropathic pain and that could have a discriminant value (Bouhassira *et al.*, 2005). Most RDEB patients complained of numbness, itching, pins and needles, burning, tingling and electric shocks sensations. These symptoms are highly suggestive of neuropathic pain.

The DN4 questionnaire was developed as a screening tool to discriminate between neuropathic and non-neuropathic pain. A score of 4 or higher suggests neuropathic pain (Bouhassira *et al.*, 2005). This questionnaire has been used in large epidemiological studies to estimate the prevalence of neuropathic pain both in the general population (Bouhassira *et al.*, 2008) and specific clinical situations (e.g. diabetic neuropathy) (Spallone *et al.*, 2012). In this study we found that over three-quarters of RDEB patients had a score of 4 or higher, and the mean score was 5.03 ± 0.43. Most patients with a definite or probable diagnosis of neuropathic pain, as defined by the updated IASP grading system (Finnerup *et al.*, 2016), scored over the cut-off value. The DN4 is a useful screening tool in the clinical setting, because of its simplicity (Haanpaa *et al.*, 2011). Performance will vary in different settings (Scholz *et al.*, 2009); in our hands in the context of RDEB it had a sensitivity of 82% and specificity of 73% for the diagnosis of neuropathic pain.

### Sensory profile in RDEB

The quantitative testing revealed that RDEB patients had reduced sensitivity to warm and cool stimuli, which is indicative of loss-of-function mediated by C and Aδ fibres. Interestingly, the loss in sensitivity was correlated with the severity of the RDEB disease.

RDEB patients also showed a number of features of gain of sensory function compared to control participants, in the form of decreased heat pain thresholds, increased mechanical pain sensitivity, and increased wind up ratio. Our healthy volunteers QST mean z-values generally lie well within the normal range of 0 ± 1.96 (compared with normative data; Vollert *et al.*, 2015). Moreover, most mean z-values per parameter are within 0 ± 0.40, except for mechanical pain sensitivity that was 1.5 ± 0.22 away from the mean reference data. Pain sensitivity differs among different races (Riley *et al.*, 2002; Cano *et al.*, 2006), and it has been reported that Latino or Hispanic minorities have higher pain sensitivities in response to experimental pain compared with non-Hispanic whites in the USA (Rahim-Williams *et al.*, 2007; Gonzalez-Duarte *et al.*, 2016). The healthy volunteers recruited in this study were from a Latino background and the normative data from the DFNS to which we compare them are mainly white European Caucasians (Rolke *et al.*, 2006). Therefore, the difference we are seeing in mechanical pain sensitivity may reflect a difference in pain sensitivity among races. Even though healthy controls had high mechanical pain sensitivity scores, RDEB patients had significantly higher mechanical pain sensitivity scores.

One-third of RDEB patients presented paradoxical heat sensations and almost one-fifth had dynamic allodynia.

It is worth mentioning that this particular QST profile is quite rare for a neuropathy in that although hyposensitivity to warm and cool detection would be quite typical (Phillips *et al.*, 2014; Themistocleous *et al.*, 2016), there is a significant group of patients that demonstrate hypersensitivity. This could be reflecting the fact that our patients not only had a painful small fibre neuropathy, but also had a persistent acute inflammatory condition that could account for a strong central sensitization component of pain.

#### RDEB patients suffer from a small fibre neuropathy

Small fibre neuropathy is a clinical syndrome in which patients present with symptoms and signs of small fibre dysfunction including spontaneous burning pain, altered thermal sensibility and autonomic symptoms due to injury selectively affecting small diameter sensory and/or autonomic axons. (Themistocleous *et al.*, 2014). For the diagnosis of small fibre neuropathy the presence of at least two abnormal results at clinical examination, QST and/or skin biopsy are required (Devigili *et al.*, 2008). Other groups propose a hierarchy of different levels of diagnostic certainty (England *et al.*, 2005; Botez *et al.*, 2008). In our study RDEB patients presented with pain of neuropathic characteristics, have a diminished capacity to detect thermal stimuli, had a reduced sympathetic skin response, and had a severe reduction in the density of intraepidermal nerve fibres. Thus, RDEB patients can be diagnosed as having a small fibre neuropathy.

The lack of functionality of small fibres and the histological findings correlate well with the severity of the disease, suggesting that the recurrent episodes of skin damage lead to injury of the small fibres in the epidermis. Neuropathy in RDEB patients was demonstrated to have a length-dependant pattern, even though blistering in RDEB has no preference to extremities. This pattern is well recognized in other causes of small fibre neuropathies such as diabetes mellitus, alcoholic and amyloid polyneuropathies, to mention a few (Said, 2007; Themistocleous *et al.*, 2016). The signs and symptoms start—and remain more pronounced—in the feet, and go on to affect more-proximal parts of the lower limbs and eventually the distal parts of the upper limbs. In a classical small fibre neuropathy large fibre sensory function is spared. In terms of clinical examination, we did not detect deficits in large fibre sensory modalities, on QST the emphasis was on small fibre dysfunction and neurophysiological studies showed there was no abnormality in motor or sensory conduction.

In this study we hypothesized that RDEB patients have a small fibre neuropathy that is secondary to chronic skin damage. An alternative explanation could be that the mutated proteins resulting in skin blistering are also important in some way for nerve fibre integrity. We investigated if other small fibres, which are not in contact with the chronically injured skin, were also affected in RDEB patients. Heart rate variability and blood pressure responses to different stimuli were preserved in RDEB patients and there was no evidence of systemic autonomic dysfunction. We then tested autonomic function of fibres innervating the skin and found impaired sympathetic skin response (a test of sympathetic sudomotor function). We also found reduced innervation of sweat glands in the dermis. These findings are consistent with selective injury to cutaneous small fibres with no evidence of generalized autonomic dysfunction.

Mutations in our cohort of patients are all located at the *COL7A1* gene, which encodes the alpha-1 chain of type VII collagen a protein restricted to the basement zone beneath stratified squamous epithelia (Sat *et al.*, 2000). Regarding expression of *COL7A1* in the nervous system very little has been reported. Expression of type VII collagen has been reported in select CNS regions such as choroid plexus epithelial cells, pineal gland and pituitary gland cell nests and cerebellar cortex while there is absent expression in peripheral nerves (Paulus *et al.*, 1995) (http://human.brain-map.org/microarray/gene/show/1285). Animals lacking *COL7A1* do not develop any abnormalities of the nervous system (Heinonen *et al.*, 1999). It is the critical role for type VII collagen in maintaining skin integrity that appears to be relevant and small sensory fibres appear uniquely vulnerable in RDEB due to the superficial location of their terminals

The exact mechanism of the injury and its length dependence is interesting and as yet not fully established. It may be direct physical severing of axons at the dermo-epidermal border. Or may be due to changes in local environment as the loss of type VII collagen can change the bioavailability of soluble proteins; an increase in TGF-β and a decrease in MMP2 have been reported (Fritsch *et al.*, 2008; Kuttner *et al.*, 2013). We do not know the timing of the reduction in IENFD—this would need prospective studies from childhood. We can’t therefore exclude developmental effects due to lack of certain factors in the skin.

### Can a dermatological condition cause neuropathic pain?

Neuropathic pain is defined as pain caused by a disease or a lesion of the somatosensory nervous system (Treede *et al.*, 2008). Traumatic, toxic-metabolic, infectious, nutritional deficits, among others can damage peripheral nerves and are well known causes of peripheral neuropathic pain. But it is yet unknown if a dermatological condition that only affects the most distal portion of sensory fibres can produce neuropathic pain.

Innervation in the skin consists of myelinated and unmyelinated fibres in the dermal plexus, and only unmyelinated fibres that cross the dermo-epidermal border reaching the epidermis. Dermatological conditions in theory could affect epidermal innervation, increasing or decreasing its density (Misery *et al.*, 2014). Indeed, histological observations have indicated that epidermal nerve fibres are present at higher densities in the skin of patients with itchy psoriasis (Nakamura *et al.*, 2003; Taneda *et al.*, 2011), lichenified atopic skin (Urashima and Mihara, 1998), photodamaged skin (Toyoda *et al.*, 2005) and in a mouse model of xerosis (Tominaga *et al.*, 2007), than in healthy individuals. On the other hand, IENFD have been shown to be reduced in conditions such as sensitive skin (Buhé *et al.*, 2016), prurigo nodularis (Schuhknecht *et al.*, 2011), and in a case report of grafted skin (Zeidler *et al.*, 2016). It could be interesting to see if these changes in skin innervation can lead to pain of neuropathic characteristics. These studies, however, do not report on the incidence of pain and the impact of primary dermatological conditions on the structure and function of sensory nerve fibres has been relatively neglected. A recent study showed that patients with pachyonychia congenita, a skin disease that presents plantar keratoderma and that exhibits pain of neuropathic characteristics, presents with abnormal mechanical detection and pain threshold (Wallis *et al.*, 2016); however, measures focusing on small fibre function such as thermal thresholds were not assessed. One recent study reported a reduction in IENFD in six patients with RDEB, but as a standardized site was not used, this could not be compared to normative data and was not related to somatosensory phenotype (Mack *et al.*, 2015). In our study we report a severe reduction of IENFD in RDEB patients compared with control subjects and normative data. Conversely, a study on one patient with junctional epidermolysis bullosa with a mutation in laminin-332 reported an increase in IENFD. Laminin-332 was shown to inhibit nerve branching and the lack of it in this patient led to an increase in IENFD (Chiang *et al.*, 2011). In this case, although the clinical phenotype bore similarity to that of our patients, the mutation is in a different gene and therefore the pathogenic mechanisms may differ.

## Conclusions

We conclude that abnormalities in the dermo-epidermal boundary in patients with RDEB lead to injury to the distal terminals of small fibres with a particular vulnerability of the longest axons. This results in small fibre dysfunction and a high incidence of neuropathic pain. Assessment and targeted treatment of neuropathic pain in this population should be instituted in order to ameliorate their disabling pain.

## Supplementary Material

Supplementary Data

## Abbreviations

BEBS: Birmingham Epidermolysis Bullosa Severity
IENFD: intraepidermal nerve fibre density
QST: quantitative sensory testing
RDEB: recessive dystrophic epidermolysis bullosa
