# Supplementary material for: Recessive dystrophic epidermolysis bullosa results in painful small fibre neuropathy
Source: Brain. 2017 Mar 28;140(5):1238–51. doi: 10.1093/brain/awx069 (PMC5405236; doi:10.1093/brain/awx069)
Supplement: Supplementary Data [file awx069_Supp.zip › brain-2016-01377-File012.pdf]

QST parameter	BEBS item						
	Involvement of nails	Area of acutely damaged skin	Acute involvement of Mucous membrane	Scarring of hands	Chronic wounds	Alopecia	Nutritional compromise
CDT	0.196	0.490	0.441	0.028	0.490	0.980	>0.5
WDT	0.000	0.098	0.490	0.000	0.006	0.006	0.062
TSL	0.056	>0.5	0.490	0.010	0.266	0.399	0.056
MDT	>0.5	>0.5	>0.5	>0.5	>0.5	>0.5	>0.5
HPT	0.029	>0.5	>0.5	0.016	0.173	>0.5	>0.5
MPS	>0.5	>0.5	>0.5	>0.5	>0.5	>0.5	>0.5
WUR	>0.5	>0.5	>0.5	>0.5	>0.5	>0.5	>0.5

Supplementary table 2: Correlation between QST parameters and different BEBS score items. Scarring of the hands correlates very well and consistently with loss of function on thermal tests (CDT, WDT, TSL, and HPT). Scarring of the hands gets worse with the repetitive cycles of blistering and regeneration and therefore reflect an active and longer lasting disease affecting the hands (and probably the lower limbs as well). This is therefore consistent with our hypothesis that small sensory fibres are sensitive to chronic injury to the epidermis which will be reflected in abnormalities in thermal parameters mediated by these fibres.

The involvement of the nails also reflects a chronic condition although it is also affected by other variables such as the exact site of the blistering (if the disease affects preferably the fingers/toes or not). The presence of chronic wounds and alopecia also correlate well with WDT, which is probably the most sensitive item in the QST to damage to intra-epidermal C-fibres. The other items of the BEBS reflect the acute phase of the disease and not so much the long-term damage of skin injury.

P-values were Bonferroni corrected for multiple comparisons.
