# Supplementary material for: Recessive dystrophic epidermolysis bullosa results in painful small fibre neuropathy
Source: Brain. 2017 Mar 28;140(5):1238–51. doi: 10.1093/brain/awx069 (PMC5405236; doi:10.1093/brain/awx069)
Supplement: Supplementary Data [file awx069_Supp.zip › brain-2016-01377-File009.pdf]

Case report of a patient with RDEB

We present the case of a 19 years old male: he was born with generalised blistering that was followed by extensive dystrophic scarring, especially on the hands and on the inner side of his left thigh. His diagnosis was first made by histology (subepidermal blistering) and confirmation was made by Sanger sequencing that showed a homozygous mutation c.5932C>T in the *COL7A1* gene (NM_000094.3). He developed pseudosyndactyly (mitten-hand deformity) as a toddler. He lost his toenails during puberty, but apart from this, his feet do not have dystrophic scarring or other deformities. He developed phimosis (aged 13), corneal scarring (aged 11), and oesophageal strictures (aged 16), all of which required corrective surgery. His sensory symptoms developed at around 8 years of age; generalised itch, which did not respond to antihistamines, was followed by a length dependant loss of thermal sensitivity. He complains of pins and needles, burning and electric shock like sensations that started on the right foot at 15 years of age and progressed to the left foot becoming more severe with time. His symptoms are worsened by cold temperature. He is on daily paracetamol (1gr every 12 hours), and occasionally takes tramadol, although this increases itching and it is not very effective in relieving his pain.

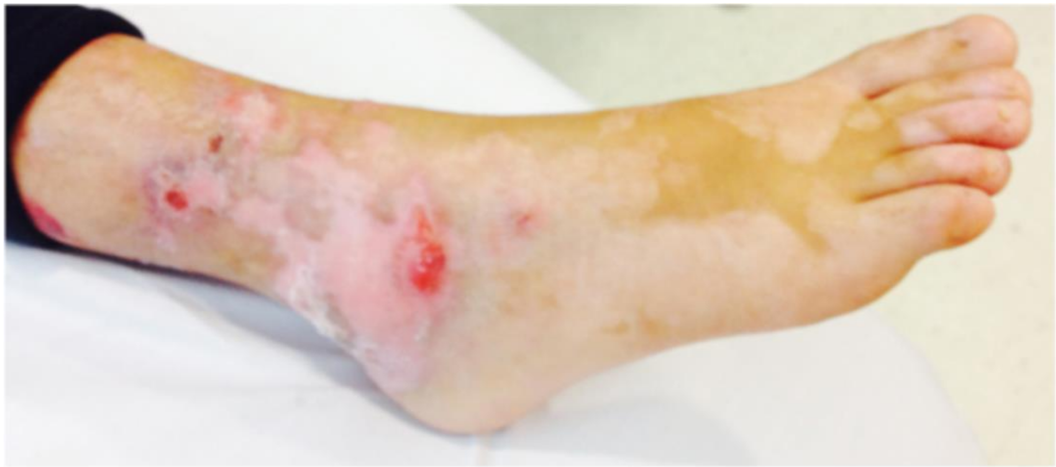

SF1. Photograph of the right foot of the patient described in the history case. Even though his foot has active blisters, scarring, and absent toenails, he doesn't have dystrophic scarring or other deformities
