# Supplementary material for: Recessive dystrophic epidermolysis bullosa results in painful small fibre neuropathy
Source: Brain. 2017 Mar 28;140(5):1238–51. doi: 10.1093/brain/awx069 (PMC5405236; doi:10.1093/brain/awx069)
Supplement: Supplementary Data [file awx069_Supp.zip › brain-2016-01377-File010.pdf]

Supplementary Figure 2

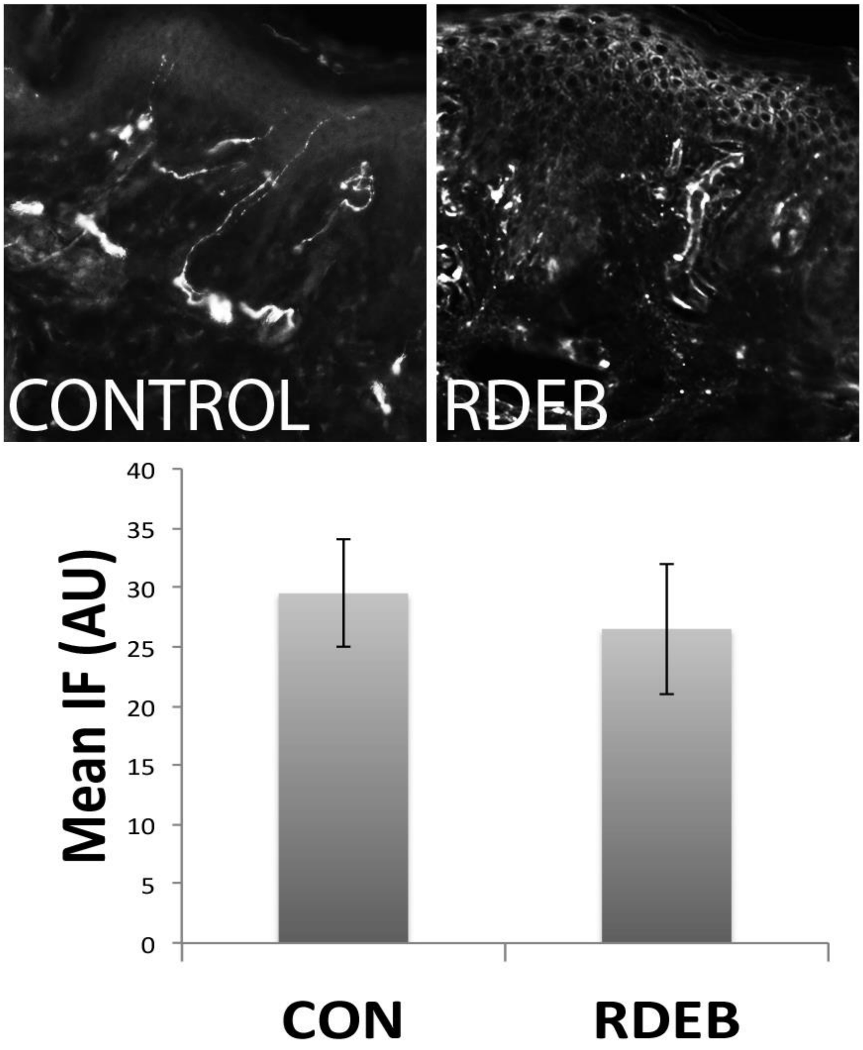

Dermal innervation: Representative sections of the skin of a control subject and a RDEB patient immunostained with PGP 9.5. Although the architecture of the skin is damaged in RDEB, innervation of the dermis remains the same, especially in blood vessels as seen in this photomicrograph. Quantification of PGP 9.5 IF signal (in arbitrary units) is kept the same in controls and RDEB (n=10)
