# Supplementary material for: Recessive dystrophic epidermolysis bullosa results in painful small fibre neuropathy
Source: Brain. 2017 Mar 28;140(5):1238–51. doi: 10.1093/brain/awx069 (PMC5405236; doi:10.1093/brain/awx069)
Supplement: Supplementary Data [file awx069_Supp.zip › brain-2016-01377-File011.pdf]

				MOTOR		SENSORY				
Age	Gender	BEBS	IENFD	Distal weakness (Y/N)	Reflexes (N/Ab)	Light touch	Vibration	JPS	Pin prick	Temperature
32	F	33.00	0.6	N	N-ua AT/P/BR	0	0	0	0	3 (LL)
18	M	48.50	0	N	N-ua BR	0	0	0	0	2 (LL)
30	F	53.00		N	N-ua AT/P/B/T/BR	0	0	0	0	3 (LL), 1 (UL)
33	F	89.00	0	N	N-ua AT/B/T/BR	0	0	0	0	3 (LL)
32	M	44.00	0	N	N-ua BR	0	0	0	0	3 (LL), 1 (UL)
16	F	57.80		N	N-ua BR	0	0	0	0	2 (LL)
19	M	45.30	0	N	N	0	0	0	0	4 (LL)
20	F	70.30	0	N	N-ua AT/P/B/T/BR	0	0	0	2 (LL)	3 (LL)
19	F	46.00	1	N	N-ua AT/B/T/BR	0	0	0	0	3 (LL)
19	M	48.00	0	N	N	0	0	0	0	1 (LL)
22	M	24.25	0	N	N-ua BR	0	0	0	0	2 (LL)
15	F	30.50	0.1	N	N-ua BR	0	0	0	0	3 (LL)
20	M	20.50	0	N	N-ua BR	0	0	0	0	3 (LL)
16	M	33.50	4.7	N	N	0	0	0	0	2 (LL)
15	M	18.00		N	N	0	0	0	0	0
20	M	22.00		N	N	0	0	0	0	0
17	M	8.50	14.5	N	N	0	0	0	0	0
17	F	8.50	4.4	N	N-ua BR	0	0	0	0	1 (LL)
16	M	12.70	1.5	N	N	0	0	0	0	0
14	F	48.50	0.1	N	N	0	0	0	0	3 (UL)
13	F	28.50	0	N	N	0	0	0	0	3 (LL), 1 (UL)
13	F	16.00		N	N	0	0	0	0	0
19	F	52.30		N	N-ua AT/P/B/T/BR	0	0	0	0	1 (LL)
20	F	26.30		N	N-ua AT/B/T/BR	0	0	0	0	1 (LL)
15	M	35.00		N	N-ua BR	0	0	0	0	1 (LL)
65	F		0	N	N	0	0	0	0	2 (LL)
57	F		0	N	N	0	0	0	0	3 (LL)
16	F		4	N	N-ua BR	0	0	0	0	1 (LL)
21	M		4.7	N	N-ua AT/P/B/T/BR	0	0	0	0	1 (LL)

Supplementary Table 1: summary of the examination findings and their relationship to RDEB severity.Data are presented as mean ± standard deviation (SD).
BEBS: Birmingham Epidermolysis-Bullosa severity score,
IENFD: intra-epidermal nerve fibre density (fibre per mm),
Reflexes: Y/N: yes or no, N/Ab: normal or absence, ua: unable to assess the following, B: Biceps (C5, C6), BR: Brachioradialis (C6), T: Triceps (C7), AT: Achilles Tendon (S1), P: Plantar,
JPS: joint position in space,
Sensory testing score: The level at which normal sensations are felt is recorded.
UL upper limbs LL lower limbs
0: up to the metacarpophalangeal joints (UL) or metatarsophalangeal joints (LL)
1: up to the wrist (UL) or ankle (LL)
2: up to the elbow (UL) or knee (LL)
3: up to the shoulder (UL) or hip (LL)
